# [Retracted] MicroRNA‑642a‑5p inhibits colon cancer cell migration and invasion by targeting collagen type I α1

**DOI:** 10.3892/or.2024.8795

**Published:** 2024-08-12

**Authors:** Xiaoguang Wang, Zhengwei Song, Biwen Hu, Zhenwei Chen, Fei Chen, Chenxi Cao

Oncol Rep 45: 933–944, 2021; DOI: 10.3892/or.2020.7905

Following the publication of this paper, it was drawn to the Editor's attention by a concerned reader that certain of the cell invasion assay data shown in Fig. 6B on p. 940, and western blot data featured in Fig. 7B on p. 942, had already appeared in previously published articles written by different authors at different research institutes. Owing to the fact that the contentious data in the above article had already been published prior to its submission to *Oncology Reports*, the Editor has decided that this paper should be retracted from the Journal. The authors were asked for an explanation to account for these concerns, but the Editorial Office did not receive a satisfactory reply. The Editor apologizes to the readership for any inconvenience caused.

